# Effects of additional electrical stimulation and *pre-rigor* conditioning temperature on the ageing potential of hot-boned bovine muscles

**DOI:** 10.5713/ajas.19.0788

**Published:** 2019-12-24

**Authors:** Prabhu Balan, Mustafa M. Farouk, Maryann Staincliffe, Adam D Stuart, Robert Kemp, Cameron Craigie

**Affiliations:** 1 Alpha-Massey Natural Nutraceutical Research Centre, Riddet Institute, Massey University, Private Bag 11 222, Palmerston North 4442, New Zealand; 2 AgResearch Ltd, Ruakura Research Centre, Hamilton 3214, New Zealand

**Keywords:** Beef, Additional Electrical Stimulation, Meat Quality, *Pre-rigor* Holding Temperature, Shear Force, Water-holding Capacity

## Abstract

**Objective:**

The aim of this study is to characterize the impact of additional electrical stimulation (AES) and various *pre-rigor* holding temperatures (for 3 h) on the ageing-potential of hot boned bovine *M. longissimus lumborum* (*LL*).

**Methods:**

Paired *LL* loins from 12 bulls were hot-boned within 40 min of slaughter, immediate AES applied and subjected to various holding temperatures (5°C, 15°C, 25°C, and 35°C) for 3 h.

**Results:**

AES did not accelerate the rate of rigor attainment, but the 3 h *pre-rigor* holding temperature did. Shear force values decreased as the *pre-rigor* holding temperatures increased. AES and holding for 3 h (at 25°C) resulted in higher water-holding capacity.

**Conclusion:**

Data confirmed that AES did not influence the various meat quality parameters in the present study, but *pre-rigor* holding temperature (25°C) alone or in combination with AES resulted in superior meat quality.

## INTRODUCTION

The red meat industry identified that improving the eating quality of beef as essential in meeting the fast-growing demands for high value beef products in discerning and highly competitive international markets [1]. It is particularly important that the industry reduce the inconsistent tenderness of beef being contributed by the so-called ‘intermediate pHu’ bull beef [2].

The ultimate pH of meat (pHu) has a considerable impact on meat quality [3]. Normal pHu samples are a bright red color that consumer’s associate with good quality and the meat tenderizes with ageing. High pHu meat results in tender but dark, firm and dry (DFD) product. In contrast to high and low pHu meat, the tenderness of intermediate pHu meat is much more inconsistent and consequently it is of poorer quality. A significant proportion of bull beef can be categorized as intermediate pHu (18%) and seasonal variation may also add to the inconsistency [4]. The underlying biochemical mechanisms related to this inconsistent tenderness issue are not fully understood. Therefore, improving the meat quality through novel meat processing systems for these classes of carcasses needs to be developed.

Varying *pre-rigor* environments generated by the application of electrical stimulation (ES) and/or *pre-rigor* chilling conditions influence the rate of glycolysis and subsequent pH decline in muscles *post mortem*. One of the major *post mortem* interventions implemented by the meat industry for enhancing meat quality attributes is carcass ES [5]. There are discrepancies among various reports on the influence of ES on meat quality. Some authors found positive effects due to ES [6,7], while others found no effects [8] and others reported negative effects [9]. Despite the differences, ES has been shown to enhance tenderness due to reduction in muscle ATP [5]. The complex interaction of pH and temperature decline in *pre-rigor* muscle has a significant role in meat tenderisation by influencing proteolytic enzyme activity, particularly μ-calpain [6]. The meat industry typically refers to this as the “pH/temperature window” as hot or cold shortening can result from over stimulation or high chilling rate before the pH has declined sufficiently. Optimal tenderness was evident when glycolysis had proceeded at an intermediate rate resulting in 6.1 pH at 3 h *post mortem* [10].

Recently, Balan et al [6] demonstrated that various *pre-rigor* holding temperatures (especially 25°C or 35°C) along with accelerated pH decline rates by low voltage electric stimulation (LVES) can have positive effects on bull *M. longissimus lumborum*. The treatments resulted in improved tenderness, reduced cook loss and increased sarcomere length. Furthermore, the treatments positively influenced the myofibrillar proteins and small heat shock proteins (sHSP) activities associated with μ-calpain activity in bull beef samples. LVES combined with the 3 h *pre-rigor* holding temperature (for ES-25 and ES-35 samples) resulted in no cold shortening (or heat induced shortening) even though the samples were aged at 1°C. Based on the above findings [6], it can be implied that there is an optimal condition (i.e. combination of LVES with 3 h *pre-rigor* holding temperature at 25°C or 35°C) for maximizing the ageing-potential for bull beef. Moreover, the results discussed above [6] from bulls which were stunned by captive bolt (carried out to avoid any electrical effects before stimulation), need to be verified (in all the three ranges of pHu) using current slaughter practices where the animals are electrical stunned and immobilized pre-slaughter.

We hypothesize that the additional electric stimulation (AES), *pre-rigor* pH, and temperature decline will positively influence the ageing-potential of hot boned bull beef muscle, particularly from intermediate pHu carcasses. Therefore, the objective of this study is to characterize the impact of AES and various *pre-rigor* holding temperatures (for 3 h) on the ageing-potential of hot boned bull beef muscle.

## MATERIALS AND METHODS

### Raw materials and processing

**Figure 1:**
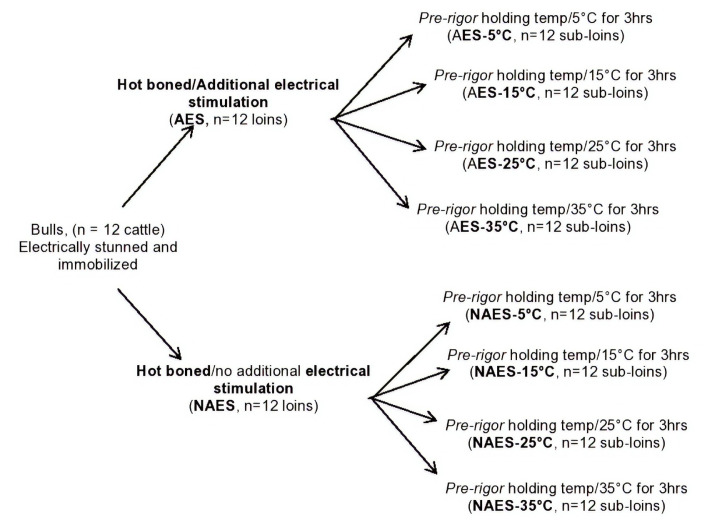
Experimental design to determine the influence of additional electrical stimulation (AES) and various *pre-rigor* holding temperature for 3 h *post mortem* of beef *M. longissimus lumborum* samples.

A total of 12 cattle (around 24-month-old bulls) were slaughtered at a New Zealand meat plant over three slaughter days. Bulls in this study were electrically stunned for Halal kill (frequency = 50 Hz, pulse width = 3.5 milliseconds, peak voltage = 583 volts) and the carcasses electrically immobilized following exsanguination. Both loins (*M. longissimus lumborum*; *LL*) from the 12 beef carcasses were hot-boned within 40 min *post mortem*. In this study, two main treatment effects i.e., AES, no additional electrical stimulation (NAES) and *pre-rigor* holding temperature (at 5°C, 15°C, 25°C, and 35°C for 3 h) and their interactions were tested giving a total of 8 different treatment combinations (Figure 1).

**Figure 2:**
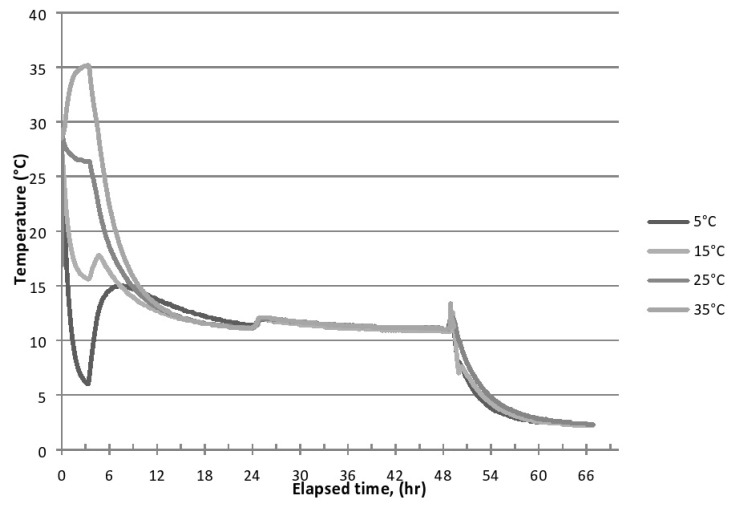
Temperature decline rates of the bull beef *M. longissimus lumborum* muscles assigned to additional electrical stimulation and various *pre-rigor* holding temperature (for 3 h) methods. The samples (in plastic bag) were then placed in boxes at 10°C for 24 h *post mortem*. Then samples were vacuum packed and aged at 10°C for next 24 h (in total 48 h *post mortem*) and further aged at 1°C until 14 days *post mortem*.

Initial pH (pH_40min_) was recorded and the *LL* from either left or right (randomly selected) side of the carcass was immediately subject to low voltage electrical stimulation (AES) for 30 S after boning (frequency = 13.3 Hz, pulse width = 5.4 milliseconds, peak voltage = 104 volts). After stimulation, pH was recorded once again. The *LL* from the other side of the carcass was not additionally electrically stimulated (NAES). Immediately after stimulation or non-stimulation, approximately 10 g of muscle was removed, snap frozen using liquid nitrogen and stored at −80°C for initial biochemical analyses. The loins were then divided into four different sub-samples, placed in plastic bags and randomly submerged in either 5°C, 15°C, 25°C, or 35°C water baths for 3 h, which was the *pre-rigor* holding temperature. A temperature probe was inserted in to the geometric center of each loin section to monitor continuous drop in temperature (Figure 2). After 3 h had elapsed, the meat/muscle samples were removed from the plastic bags; their pH measured, and further sampled for biochemical analysis. The loin samples were transferred to the AgResearch laboratory where they were vacuum packed and aged at 10°C for 48 h. After 48 h sampling and measurements, loins were again vacuum packed and aged at 1°C until 14 days *post mortem*.

### pH

pH of the LL samples was measured in duplicate by inserting a calibrated pH probe (Hanna HI99163 pH meter with a FC232D combined pH/temperature probe, HANNA instruments, Woonsocket, RI, USA) directly into the muscle at 40 min (before and after stimulation), 3 h, 6 h, 24 h, 48 h, and 14 days post mortem respectively.

### Shear force

The *LL* samples were cooked in a water bath set at 99°C (controlled by a Digi-Sense scanning temperature logger [Eutech Instruments Pte Ltd., Singapore] with a thermocouple positioned into the center of each sample) to an internal temperature of 75°C. After cooking, the samples were transferred to ice-water slurry for at least 10 min. Shear force was measured by determining the force required to shear through a 10 mm×10 mm cross section sample at right angles to the fiber axis using the MIRINZ tenderometer [11]. Ten replicates were measured for each pre-cooked sample. The results were expressed as shear force (kgF) and final values of peak shear force were calculated as an average from the 10 replicates.

### Cooking loss

*LL* samples were weighed before and after cooking. The cook loss was calculated as weight before cooking minus weight after cooking and expressed as a percentage of the pre-cooked weight [6].

### Purge and drip loss (water holding capacity)

The *LL* sections were weighed prior to vacuum-packaging to obtain initial weight for the purge loss measurement. After the assigned storage time, the samples were removed from the vacuum bags, patted dry on paper towels and reweighed (final weight) to determine purge loss as the difference between initial weight and final weight expressed as percentage of initial weight.

Drip loss was measured after each assigned storage (48 h and 14 days) following the procedure of Balan et al [12]. A sample (about 50 g) of meat with any visible fat and connective tissue removed was weighed and then placed in plastic ‘onion bag’ netting and suspended by a hook within a closed container. After placing the container for 48 h at 4°C, the sample was blotted dry and then reweighed. The drip loss was calculated as weight lost expressed as a percentage of the original sample weight.

### Color

The cuts (48 h *post mortem*) for color measurements were placed in Cryovac food grade trays (Cryovac TQD-0900; 22.5 cm×17.3 cm×4.1 cm; CRYOVAC, Sealed Air Corporation, Duncan, SC, USA) with the cross sectional side up and then sealed with oxygen barrier film using Cryovac LID 1050 (CRYOVAC, Sealed Air Corporation, USA) into HiOx-MAP. A high-oxygen modified atmosphere (80% O_2_/ 20% CO_2_, certified standard within ±5%, BOC GASES; Hamilton, New Zealand) was accomplished using a ROSS Inpack Junior A10 Packaging Machine (Ross industries packaging division, Midland, VA, USA) by applying vacuum, then flushing the package with the gas mixture and sealing. The gas mixture composition inside each pack was checked before opening with a PBI Dansensor, CheckPoint handheld gas analyzer (Ringsted, Denmark) by piercing the top layer and reading the oxygen and carbon dioxide levels. The packaged cuts were displayed for 7 days at 3°C±1°C under continuous fluorescent natural white light (2,800 lx, color rendering index = 82, color temperature = 4,000 K; Osram, Auckland, New Zealand).

On days 1, 4, and 7 of simulated retail display under light, the cut cross sectional meat surface was measured using a Minolta Color Meter (Illuminant D65, 1 cm diameter aperture, 10° standard observer; CR-300; Konica Minolta Photo Imaging Inc., Tokyo, Japan) for color using the CIE L* a* b* color space. Calibration was performed by using a standard white tile prior to the color measurement. L* (lightness), a* (redness), and b* (yellowness) values were used to calculate chroma ([a*^2^+b*^2^]^1/2^) and hue angle ([b*/a*]^tan-1^) [13]. The surface meat color was scanned with the color meter covered with the same film after removing the plastic film on the top of the HiOx-MAP tray.

### Statistical analysis

All statistical analysis was performed using Genstat 16th edition [14]. The pH fall was analyzed using analysis of variance (ANOVA) where side within animal was the blocking variable and the treatment variables were temperature, electric stimulation, time and all possible 2- and 3-way interactions. Firstly, data was analyzed excluding pHu using ANOVA. The shear forces, cook loss, drip and purge loss (for 48 h and 14 days) data were the dependent variables. For the above mentioned analysis, side within animal was included as a blocking variable and the treatment variables were temperature (5°C, 15°C, 25°C, and 35°C) and additional low voltage electrical stimulation and their interaction. Then these variables were re-analyzed including pHu as a treatment variable, where pHu was split into three levels: low (5.4 to 5.79); intermediate (5.8 to 6.19) and high (>6.2). The initial pH for the animal was also included in the treatment structure. The color variables were analyzed using repeated measure ANOVA across 1, 4, and 7 days. Side within animal was included as a blocking variable and the treatments were temperature, AES, time and their 2- and 3-way interactions. Then this analysis was re-run including pHu (low, intermediate, and high) as a treatment and all 2-, 3-, and 4-way interactions. Least squares means for each attribute were separated using least significant differences (F test, p<0.05).

## RESULTS AND DISCUSSION

### Ultimate pH of meat samples

In this study, out of the 12 bull carcasses used, six had high (>6.2), three intermediate (5.8 to 6.2) and three low pHu (5.4 to 5.79) (Table 1).

### Temperature and pH decline

AES had no effect on pH decline (p>0.05). AES did not result in an immediate fall in pH as expected (Table 1) when compared to our previous findings [6,7]. This could be due to the electrical inputs into the carcasses from head-only electrical stunning and immobilization that masked any effect of AES. *Pre-rigor* holding temperature (for 3 h) and time-point significantly influenced pH fall (p<0.001). Generally, the highest pH decline was at 6 h *post mortem* except for 35°C samples where the highest pH fall was at 3 h *post mortem*. It is well documented that high *pre-rigor* temperature accelerates *post mortem* pH decline [6,7,15]. In comparison to our previous trial in which no electrical stunning or immobilization was used [6,7], in the present study, AES-35 samples muscle pH (in low pHu samples, Table 1) declined only by ΔpH 0.17 pH units and 0.75 soon after ES and 3 h of holding when compared to 0.43 pH units and 1.37 in the previous trial [6]. This loss of AES effect was due to the head-only electrical stunning and immobilization procedure carried out before AES and continued to be low when compared to rest of the samples (Table 1).

### Shear force

The AES treatment applied to the hot-boned loin samples did not result in significant (p>0.05) changes in shear force values (Table 2). At 48 h *post mortem*, shear force values were not significantly different for the AES samples when compared to the NAES samples (Table 2). The 3 h *pre-rigor* holding temperature had significant (p = 0.042) effect on reducing the shear force values, where AES-35 samples had most tender meat when compared to the AES-5. For both AES and NAES shear force values decreased as the temperature increased. This decrease was greatest for the AES samples and the shear force for AES-5 is higher than AES-35.

The trend was not observed in the shear force values at 14 days *post mortem* (Table 2), where there was no significant difference between the shear force values for AES and NAES samples (p>0.05). However, in high pHu samples the 3 h *pre-rigor* holding temperature had a significant effect on the shear force values recorded at 48 h *post mortem* (p = 0.046). AES-35 samples had the least shear force values (less than 7 kgF), when compared to the AES-5. At 14 days *post mortem*, there was no significant difference for the shear force values among the three pHu categories due to the additional stimulation and 3 h *pre-rigor* holding temperature. Interestingly, at 48 h *post mortem* when compared to low and high pHu samples, most of the intermediate pHu samples (both AES and NAES) had higher shear force values (Taking 11 kgF as the upper limit for acceptable cooked meat tenderness [16]). However, after 14 days of ageing this observed effect was lost as all the samples (intermediate pHu) were tender (less than 7.5 kgF) except the ES-35 samples which was ~8.8 kgF. This observation supports previous findings, that the increased toughness of meat with a ultimate pH between 5.8 to 6.19 is due to slow rate of tenderization [4,17].

**Figure 3:**
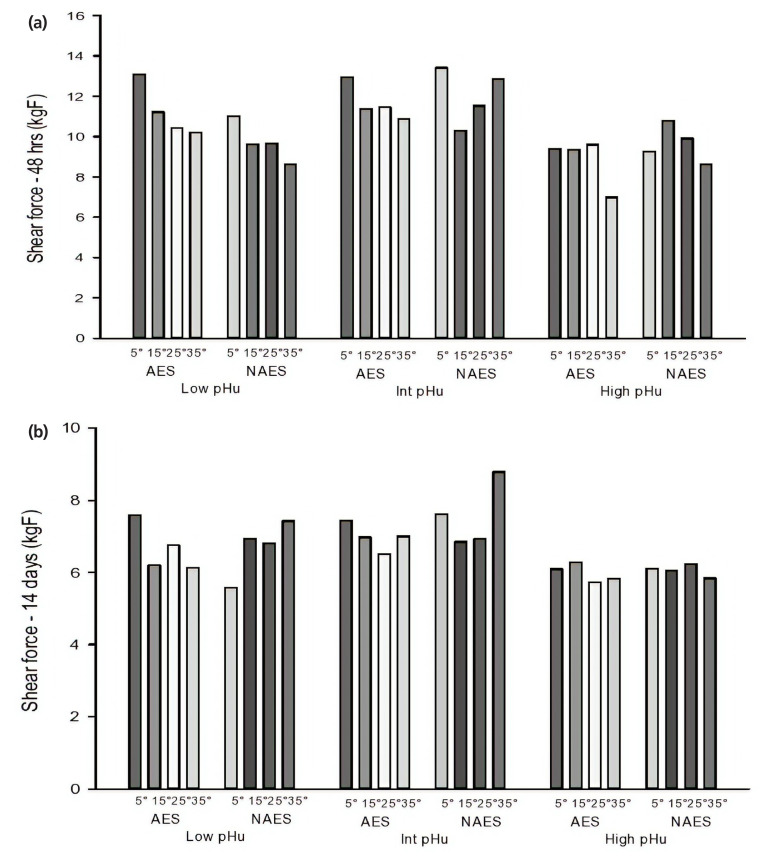
(a) Effect of AES and various *pre-rigor* holding temperature (for 3 h) methods on shear force (48 h *post mortem*) of low, intermediate and high pHu beef *M. longissimus lumborum* samples. AES, additional electrical stimulation; NAES, no additional electrical stimulation. Low pHu (n = 3), Int (intermediate) pHu (n = 3), and high pHu (n = 6). Treatment effect [AES vs ES] (p>0.05); pH effect (p>0.05); temperature effect (p = 0.046); interaction effect (p>0.05, standard error of difference [SED] = 1.76). (b) Effect of AES and various *pre-rigor* holding temperature (for 3 h) methods on shear force (14 days *post mortem*) of low, intermediate and high pHu beef samples. Low pHu (n = 3), Int (intermediate) pHu (n = 3), and high pHu (n = 6). Treatment effect [AES vs ES] (p>0.05); pH effect (p>0.05); temperature effect (p>0.05); interaction effect (p>0.05, SED = 0.63)

Earlier findings have suggested that *pre-rigor* temperature had the most dominant effect on the shear force values [18]. It was found that samples held at 38°C had significantly higher shear force values when compared to 15°C samples. However, in this study the effect was evident only in the intermediate pHu beef samples (Figure 3a, 3b). The difference in the findings of the two studies may be due to the long storage at high temperature (38°C) by Kim et al [18] that would have caused the protein to denature. However, in the present study the samples were held at 35°C only for 3 h in *pre-rigor* compared to the 24 h at 38°C in the former study.

### Cook loss

**Figure 4:**
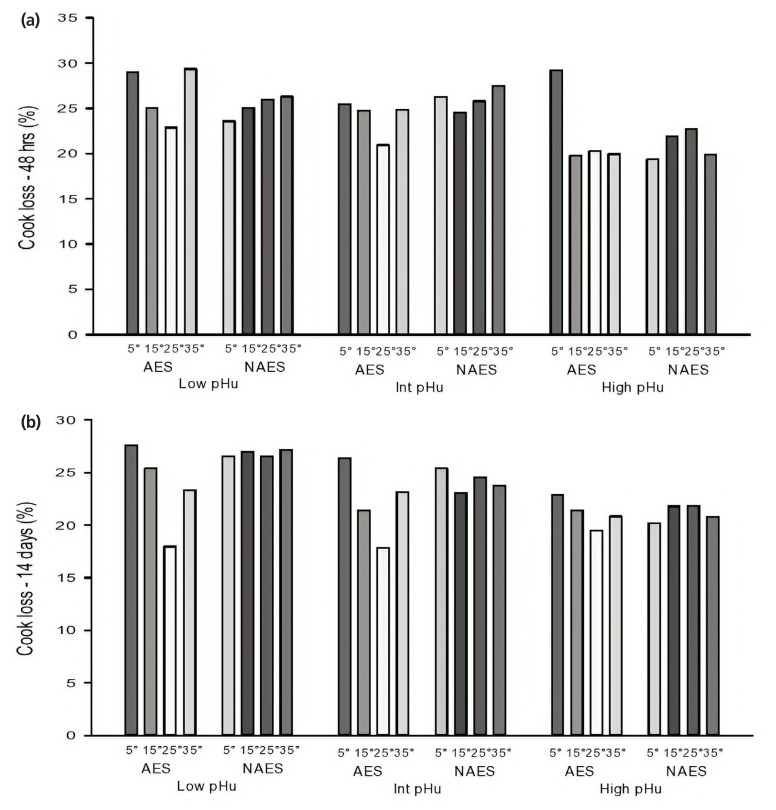
(a) Effect of AES and various *pre-rigor* holding temperature (for 3 h) methods on cook loss (48 h *post mortem*) of low, intermediate and high pHu beef *M. longissimus lumborum* samples. AES, additional electrical stimulation; NAES, no additional electrical stimulation. Low pHu (n = 3), Int (intermediate) pHu (n = 3), and high pHu (n = 6). Treatment effect [AES vs ES] (p>0.05); pH effect (p = 0.008); temperature effect (p = 0.039); Interaction effect (stimulation×temperature) (p<0.001, standard error of difference [SED] = 1.79). (b) Effect of AES and various *pre-rigor* holding temperature (for 3 h) methods on cook loss (14 days *post mortem*) of low, intermediate and high pHu beef samples. Low pHu (n = 3), Int (intermediate) pHu (n = 3), and high pHu (n = 6). Treatment effect [AES vs ES] (p>0.05); pH effect (p = 0.05); temperature effect (p>0.05); Interaction effect (stimulation×temperature) (p = 0.015, SED = 1.87).

At the sampling points of 48 h and 14 days *post mortem*, there was a significant interaction (p<0.01) effect of AES and *pre-rigor* holding temperature on cook loss for the AES and NAES (Table 2). Previous findings have shown that cooking loss was not affected by ES [6,7]. Among the AES samples, the cook loss was greatest for AES-5 and lowest for AES-25. The cook loss was significantly higher for AES-5 when compared to at all the other *pre-rigor* holding temperatures (p< 0.05) [6,7]. Contrary to our results, earlier findings have shown that higher *pre-rigor* temperature results in higher cook loss [19]. The difference between the findings of the two studies may be due to the long storage at different temperature (15°C or 35°C) until rigor. Generally cooking loss was lowest for the high pHu groups at both time points (p = 0.008 and 0.055 for 48 h and 14 days *post mortem* respectively) (Figure 4a, 4b).

### Purge loss

**Figure 5:**
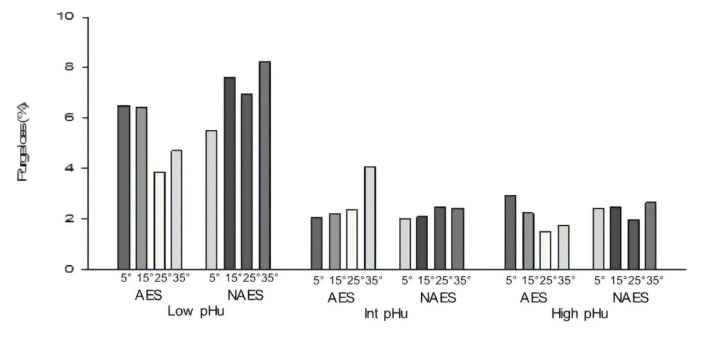
Effect of AES and various *pre-rigor* holding temperature (for 3 h) methods on purge loss (14 days *post mortem*) of low, intermediate and high pHu beef *M. longissimus lumborum* samples. AES, additional electrical stimulation; NAES, no additional electrical stimulation. Low pHu (n = 3), Int (intermediate) pHu (n = 3), and high pHu (n = 6). Treatment effect [AES vs ES] (p>0.05); pH effect (p<0.001); temperature effect (p = 0.014); interaction effect (stimulation×temperature) (p = 0.006); (pH×temperature) (p = 0.011); (stimulation×temperature×pH) (p = 0.002, standard error of difference = 0.63).

The influence of AES on purge loss was dependent on pre-rigor holding temperature (Table 2; p = 0.03). Previous findings have shown that ES had no effect on purge loss [18]. Among, the AES samples, AES-25 had significantly lower purge than AES-5. However, in NAES samples there was no significant (p> 0.05) difference in the purge loss for the different temperatures. Purge loss was significantly influenced by pHu (Figure 5; p<0.05). Generally, the purge loss was greater for the low pHu group than for the high pHu group. Interestingly, for the low pHu group (among the AES samples) the purge loss was least for 25°C than 5°C and 15°C and among NAES samples, purge loss was highest for 35°C than for 5°C and there was no difference for the intermediate and high pHu groups [18].

### Drip loss

AES had no effect on drip loss at 48 h *post mortem* (Table 2; p>0.05), but the 3 h *pre-rigor* holding temperature did. For both AES and NAES samples, drip loss was higher for 5°C and 35°C and lower for 15°C and 25°C. Our findings are somewhat similar to the earlier findings [18], which reported that drip loss was higher for the meat held at 38°C *pre-rigor* when compared to the 15°C beef samples. After 14 days of ageing, (Table 2) drip loss was significantly influenced by AES, *pre-rigor* holding temperature and their interaction effect (p = 0.029). These results are contradictory to the earlier finding [18], which reported that additional stimulation did not influence the drip loss values. The difference between the current findings and the previous findings may be due to different *pre-rigor* conditions.

For NAES samples there was no significant difference in drip loss between the various *pre-rigor* holding temperatures. Among AES samples, the samples held at 25°C for 3 h had the lowest drip loss compared to the others (p<0.05). At rigor and during the early *post rigor* period, the drip loss value of AES-15 samples was lower compared to AES-35 samples. However, as the ageing (14 days *post mortem*) progressed the drip loss values of the AES-15 samples increased to greater than AES-35 [20].

**Figure 6:**
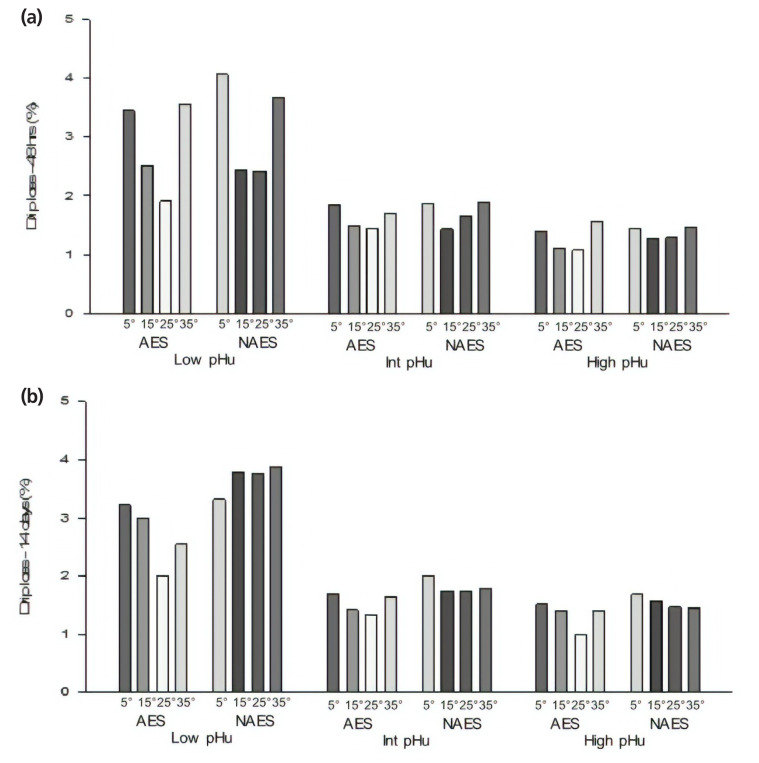
(a) Effect of AES and various *pre-rigor* holding temperature (for 3 h) methods on drip loss (48 h *post mortem*) of low, intermediate and high pHu beef *M. longissimus lumborum* samples. AES, additional electrical stimulation; NAES, no additional electrical stimulation. Low pHu (n = 3), Int (intermediate) pHu (n = 3), and high pHu (n = 6). Treatment effect [AES vs ES] (p>0.05); pH effect (p<0.001); temperature effect (p<0.001); interaction effect (pH×temperature) (p = 0.015, standard error of difference [SED] = 0.17). (b) Effect of AES and various *pre-rigor* holding temperature (for 3 h) methods on drip loss (14 days *post mortem*) of low, intermediate and high pHu beef samples. Low pHu (n = 3), Int (intermediate) pHu (n = 3), and high pHu (n = 6). Treatment effect [AES vs ES] (p<0.008); pH effect (p<0.001); temperature effect (p = 0.003); interaction effect (stimulation×temperature) (p = 0.015); (stimulation×temperature×pH) (p = 0.036, SED = 0.27).

Drip loss was significantly influenced by pHu (Figure 6a, 6b; p<0.001). In the intermediate and high pHu groups there was no significant difference in the drip loss for the various *pre-rigor* holding temperatures. Whereas for the low pHu samples, the drip loss for 15°C and 25°C is lower than for 5°C and 35°C. It is well documented that the formation of drip is thought to be as a result of shrinkage of the myofibrils due to the pH fall *post mortem* and the denaturation of protein such as myosin, which occurs when a low pH and a high *pre-rigor* temperature environment is created [21]. In this study, based on the pH (Table 1) and temperature (Figure 2) data, AES-35 reached low value of pH (<5.9), whereas the sample temperature was maintained at 35°C for 3 h. The pH decline after death was much slower in AES-25 and AES-15 samples. The observed pH and *pre-rigor* holding temperature conditions in the AES-35 are severe enough to cause the excess drip loss. Interestingly, after 14 days of ageing, drip loss was significantly influenced by AES, pHu, *pre-rigor* holding temperature and their interaction effect (p<0.05) (Figure 6b). However, for AES samples, there was significantly higher drip loss observed at 5°C than at 25°C in the low pHu group only. As expected, drip loss was greatest in low pHu group when compared to other pHu groups. However, the AES-25 treatment of the low pHu group had almost similar drip loss values to other samples from high and intermediate pHu groups. This observation requires further investigation to validate the finding in order to establish whether it is a feasible method of reducing drip loss in low pHu beef. It was proposed that there was a clear relationship between drip loss and shear force values (r^2^ = 0.70) which was observed in this study [20].

### Water holding capacity

**Figure 7:**
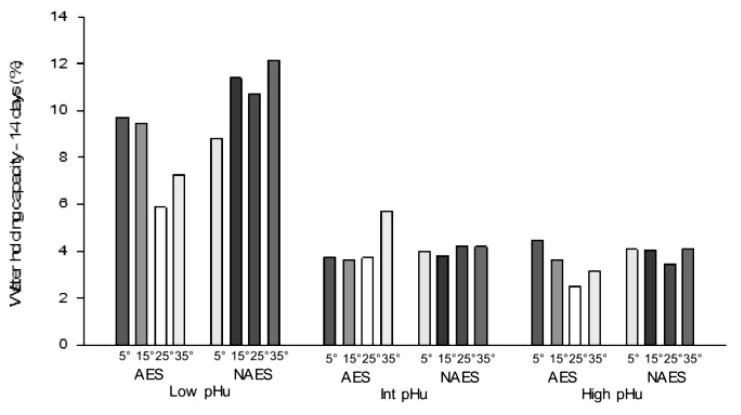
Effect of AES and various *pre-rigor* holding temperature (for 3 h) methods on water holding capacity (% loss) (14 days *post mortem*) of low, intermediate and high pHu beef *M. longissimus lumborum* samples. AES, additional electrical stimulation; NAES, no additional electrical stimulation. Low pHu (n = 3), Int (intermediate) pHu (n = 3), and high pHu (n = 6). Treatment effect [AES vs ES] (p = 0.02); pH effect (p<0.05); temperature effect (p = 0.001); interaction effect (stimulation×temperature) (p = 0.002); (stimulation×temperature×pH) (p = 0.021, standard error of difference = 0.16).

The water holding capacity (WHC % loss) data presented in this study is defined as the sum of the purge and the drip loss values, where lower values denote higher WHC and vice versa. The interaction between AES and *pre-rigor* holding temperature applied to the hot-boned loin samples significantly influenced the WHC (Table 2; p = 0.009) as was previously reported [18]. A significant increase in WHC was observed for AES-25 when compared to the NAES-25 treatment. There was no significant difference in WHC between NAES samples due to the various *pre-rigor* holding temperatures, whereas there was significant difference between AES-25 compared to AES-5. WHC was significantly influenced by pHu (p< 0.05). Among the three pHu groups, the low pHu treatment had the least WHC for each *pre-rigor* holding temperature (Figure 7). By contrast, in the AES treatments, there was significantly less WHC for AES-5 and AES-15 for the low pHu treatment when compared to high and intermediate pHu treatments. As expected, the low pHu samples (AES-25 and AES-35) had significantly lower WHC when compared to high pHu samples at the same temperatures.

Among the NAES samples, there was no significant difference between the various *pre-rigor* holding temperatures for each of the pHu samples. However, AES (in high and low but not in intermediate pHu) significantly increased the WHC for AES-25 when compared to AES-5 treatments. These findings contradicted earlier findings [22], which reported low pH and high temperature condition in post mortem muscle reduced WHC due to the denaturation of muscle proteins such as myosin. In the current study, AES and suitable *pre-rigor* holding temperature such as AES-25 based can override the negative effects of ES [20].

### Color stability

There are inconsistent reports in the literature on the effects of ES on color stability. Some studies found no color difference due to ES treatment [23,24]. In general, it is documented that ES significantly improves lean color appearance, possibly by ensuring more complete *post mortem* glycolysis within 24 h [25,26]. Overall, the AES applied to the hot-boned loin samples did not influence (p>0.05) color stability (Table 3; p>0.05).

The various *pre-rigor* holding temperatures and time (display days) did influence (p<0.05) color stability but no significant difference was found due to AES (p>0.05). When compared to AES-5, AES-35 samples had significantly lower L* values (indicator of lightness) regardless of time (display days) or stimulation and AES-35 samples had the least L* values at day 1 and 4. Farouk and Swan [27], found 24 h *post mortem* samples held at 35°C had significantly higher L* values when compared to 5°C samples and it was suggested that high rigor temperature caused protein denaturation. In this study, samples were exposed to high *pre-rigor* temperature only for 3 h. The values of red (a* values), yellow (b*) color, hue angle (indication of discoloration) and chroma (indication of saturation of color or vividness) values decreased as display days increased. The values were significantly lower on day 7 when compared to day 1. Our results are contradictory to the recent findings of Kim et al [15], who reported that hot-boned beef loins within 30 min *post mortem* kept in a 38°C water bath during the *pre-rigor* seemed to have greater L*, a*, hue and chroma angle values at 1 day *post mortem* compared with the loins held at 15°C, regardless of ES of the beef carcass. The discrepancy between the two studies may have been due to the long storage at high temperature (at 38°C) until rigor in their studies which had resulted in the protein denaturation and myofibrillar lattice shrinkage [24,28].

For a* and b* values, there was a significant interaction effect between display day and pHu group (Table 4). For a* there was also a significant interaction effect between 3 h *pre-rigor* holding temperature and pHu group. Among the low and intermediate pHu groups, day 1 a* values were the highest when compared to days 4 and 7. In the high pHu group, there was no significant difference due to the duration of display, except for the observation that samples held at 35°C for 3 h *pre-rigor* had a lower a* values for day 4 and 7 when compared to day 1. Similarly, day 4 samples that were held at high *pre-rigor* temperature (i.e. 35°C) had lower a* values when compared to other *pre-rigor* temperature treatments. There was a significant interaction effect between display day and pHu interaction on Hue angle. For chroma angle, the display day and *pre-rigor* temperatures are influenced by the pHu group (Table 4).

## CONCLUSION

The results from the current study demonstrate that AES applied, after electrical stunning and Halal slaughter did not influence the various meat quality parameters of bull beef *M. longissimus lumborum* except the drip loss (only at 14 days of ageing). However, the *pre-rigor* holding temperature (i.e. 25°C) alone or in combination with AES resulted in more tender meat, less cook loss, decreased purge and drip loss and increased WHC in bull beef samples.

## Figures and Tables

**Table 1:** Effect of additional electrical stimulation and *pre-rigor* holding temperature (for 3 h) on rate of pH fall in low, intermediate and high pHu beef *M. longissimus lumborum* samples

Items	AES				NAES			
	
	5°C	15°C	25°C	35°C	5°C	15°C	25°C	35°C
Low pHu (average)								
Before ES	6.63	6.63	6.63	6.63	6.59	6.59	6.59	6.59
After ES	6.46	6.46	6.46	6.46	6.59	6.59	6.59	6.59
3 h	6.41	6.31	6.13	5.88	6.46	6.28	6.15	6.04
6 h	6.19	6.06	5.8	5.66	6.17	6.01	5.87	5.56
24 h	5.67	5.6	5.64	5.56	5.64	5.58	5.61	5.58
48 h	5.61	5.59	5.6	5.61	5.59	5.58	5.62	5.61
14 days	5.72	5.72	5.68	5.69	5.74	5.69	5.68	5.70
Intermediate pHu (average)								
Before ES	6.62	6.62	6.62	6.62	6.70	6.70	6.70	6.70
After ES	6.47	6.47	6.47	6.47	6.70	6.70	6.70	6.70
3 h	6.52	6.45	6.29	6.02	6.54	6.34	6.42	6.17
6 h	6.34	6.31	6.21	5.96	6.43	6.34	6.2	5.99
24 h	6.19	6.15	6.09	6.01	6.12	6.09	6.03	5.94
48 h	6.07	6.09	6.06	5.96	5.96	6.05	5.97	5.93
14 days	6.13	6.14	6.08	6.07	6.04	6.08	6.02	6.02
High pHu (average)								
Before ES	6.56	6.56	6.56	6.56	6.55	6.55	6.55	6.55
After ES	6.57	6.57	6.57	6.57	6.55	6.55	6.55	6.55
3 h	6.64	6.48	6.44	6.38	6.73	6.50	6.41	6.35
6 h	6.48	6.40	6.35	6.36	6.47	6.49	6.40	6.35
24 h	6.40	6.49	6.45	6.42	6.44	6.32	6.51	6.39
48 h	6.44	6.48	6.47	6.42	6.50	6.36	6.40	6.41
14 days	6.52	6.46	6.46	6.32	6.49	6.47	6.37	6.35

n = 3 (low pHu, 5.4 to 5.79); n = 3 (intermediate pHu, 5.8 to 6.19); n = 6 (high pHu, >6.2).

AES, additional electrical stimulation; NAES, no additional electrical stimulation.

**Table 2:** Effect of additional electrical stimulation and *pre-rigor* holding temperature (for 3 h) on shear force, cook loss and water-holding capacity of beef *M. longissimus lumborum* samples

Items	AES				NAES				SED	p-value		
		
	5°C	15°C	25°C	35°C	5°C	15°C	25°C	35°C		Trt	Temp	Interaction
Shear force (kgF) 48 h *pm*	11.21	10.32	10.27	8.76	10.73	10.37	10.25	9.69	0.63	>0.05	0.042	>0.05
Shear force (kgF) 2 wk *pm*	6.81	6.44	6.18	6.20	6.35	6.47	6.55	6.98	0.22	>0.05	>0.05	>0.05
Cook loss (%) 48 h *pm*	28.20	22.32	21.08	23.51	22.13	23.34	24.28	23.39	0.89	>0.05	>0.05	>0.05
Cook loss (%) 2 wk *pm*	24.93	22.39	18.68	22.05	23.07	23.40	23.71	23.15	0.69	>0.05	0.049	0.009
Purge loss (%) 2 wk *pm*	2.03	1.56	1.37	2.10	2.20	1.61	1.67	2.13	0.17	>0.05	>0.05	0.03
Drip loss (%) 48 h *pm*	1.99	1.80	1.33	1.75	2.17	2.17	2.11	2.15	0.17	0.015	0.005	0.029
Drip loss (%) 2 wk *pm*	3.61	3.29	2.31	3.07	3.08	3.67	3.34	3.99	0.17	<0.001	<0.001	0.015
WHC (%) 2 wk *pm*	5.59	5.09	3.64	4.82	5.25	5.84	5.45	6.14	0.16	<0.05	0.002	0.021

AES, electrical stimulation; NAES, no additional electrical stimulation; SED, standard error of difference; WHC, water-holding capacity; 2 wk, 2 weeks; *pm*, *post mortem* (n = 12).

**Table 3:** Effect of AES and various *pre-rigor* holding temperature (for 3 h) methods on the color stability (48 h *post mortem*) of beef *M. longissimus lumborum* samples

Items	AES				NAES				SED	p-value			
		
	5°C	15°C	25°C	35°C	5°C	15°C	25°C	35°C		Trt	Temp	Day	Interaction
L^*^ value													
day 1	41.54	40.75	41.08	40.19	41.60	41.76	40.91	40.16	0.407	>0.05	<0.001	<0.001	>0.05
day 4	41.43	40.80	40.68	40.45	41.21	41.22	40.72	40.11					
day 7	40.89	40.43	40.50	39.53	40.29	40.33	40.41	40.08					
a^*^ value													
day 1	19.59	20.51	19.50	19.86	20.02	20.22	19.91	19.31	0.530	>0.05	0.017	<0.001	>0.05
day 4	17.71	18.25	17.51	16.49	17.57	17.96	17.20	16.87					
day 7	16.51	16.52	15.98	16.08	16.53	16.63	16.14	15.27					
b^*^ value													
day 1	7.01	7.57	6.92	7.05	7.22	7.41	7.01	6.71	0.279	>0.05	0.008	<0.001	>0.05
day 4	6.01	6.20	5.77	5.30	5.90	6.09	5.58	5.46					
day 7	5.39	5.31	5.04	5.16	5.37	5.36	5.15	4.75					
Hue angle													
day 1	19.70	20.21	19.50	19.54	19.82	20.11	19.42	19.13	0.401	>0.05	0.006	<0.001	>0.05
day 4	18.68	18.70	18.19	17.69	18.44	18.64	17.96	17.80					
day 7	17.97	17.72	17.39	17.67	17.92	17.87	17.59	17.08					
Chroma value													
day 1	20.81	21.87	20.70	21.08	21.29	21.54	21.11	20.44	0.585	>0.05	0.014	<0.001	>0.05
day 4	18.72	19.29	18.44	17.32	18.54	18.98	18.09	17.74					
day 7	17.38	17.36	16.77	16.89	17.39	17.48	16.95	16.01					

AES, electrical stimulation; NAES, no additional electrical stimulation; SED, standard error of difference; Trt, treatment effect [AES vs NAES], n = 12.

**Table 4:** Effect of AES and various *pre-rigor* holding temperature (for 3 h) methods on the lightness, redness and yellowness (48 h *post mortem*) of low, intermediate and high pHu beef *M. longissimus lumborum* samples

Items		AES				NAES			
	
		5°C	15°C	25°C	35°C	5°C	15°C	25°C	35°C
L^*^ value									
Low pHu	Day 1	43.18	42.35	42.80	42.00	43.33	42.87	42.11	41.85
	Day 4	43.06	42.67	42.05	42.61	42.97	42.66	42.64	41.90
	Day 7	42.16	41.90	42.61	42.14	42.26	41.89	41.86	41.87
Intermediate pHu	Day 1	42.03	41.02	42.25	40.63	42.41	42.59	41.56	41.04
	Day 4	42.45	41.14	42.08	39.90	41.79	42.65	40.36	38.97
	Day 7	40.51	41.02	41.22	39.32	40.08	40.83	40.70	39.52
High pHu	Day 1	40.57	39.87	39.83	39.15	40.46	40.94	40.10	39.03
	Day 4	40.29	39.76	39.53	39.55	40.14	40.02	39.89	39.60
	Day 7	40.39	39.50	39.20	38.29	39.38	39.39	39.59	39.38
a^*^ value									
Low pHu	Day 1	19.36	21.86	21.41	21.3	21.09	21.19	20.45	21.42
	Day 4	16.61	18.47	17.93	18.7	17.03	18.23	16.68	18.97
	Day 7	16.22	17.62	16.47	17.9	16.5	16.44	16.06	17.5
Intermediate pHu	Day 1	20.84	21.55	20.36	20.7	21.52	20.57	21.73	19.38
	Day 4	18.67	18.83	17.99	17.1	18.75	18.34	18.72	17.11
	Day 7	17.52	17.47	16.03	16.9	17.56	16.72	17.36	15.26
High pHu	Day 1	19.3	19.5	18.26	18.9	18.98	19.63	19.04	18.23
	Day 4	17.95	17.95	17.14	15.2	17.44	17.71	16.96	15.73
	Day 7	16.32	15.65	15.72	14.9	16.2	16.69	15.78	14.17
b^*^ value									
Low pHu	Day 1	6.84	8.3	8.05	7.81	7.57	7.86	7.23	7.84
	Day 4	5.85	6.76	6.37	6.52	5.87	6.6	5.6	6.89
	Day 7	5.78	6.36	5.89	6.35	5.73	5.7	5.49	6.28
Intermediate pHu	Day 1	7.74	8.15	7.27	7.43	8.03	7.37	7.59	6.65
	Day 4	6.58	6.5	6.01	5.58	6.46	6.11	6.34	5.46
	Day 7	5.94	5.66	4.92	5.51	5.76	5.16	5.67	4.67
High pHu	Day 1	6.86	7.02	6.24	6.55	6.78	7.2	6.71	6.17
	Day 4	5.91	5.82	5.39	4.6	5.74	5.83	5.32	4.75
	Day 7	5.01	4.66	4.65	4.45	5.07	5.26	4.8	4.01
Hue angle									
Low pHu	Day 1	19.51	20.80	20.63	20.20	19.75	20.32	19.52	20.17
	Day 4	19.2	20.02	19.51	19.27	18.92	19.85	18.62	20.03
	Day 7	19.42	19.88	19.51	19.56	19.08	19.13	18.77	19.8
Intermediate pHu	Day 1	20.41	20.71	19.7	19.76	20.5	19.75	19.31	18.96
	Day 4	19.36	19.04	18.48	18.04	19.01	18.31	18.72	17.69
	Day 7	18.73	17.94	16.98	18.1	18.14	17.12	18.09	17.01
High pHu	Day 1	19.56	19.74	18.86	19.13	19.62	20.13	19.41	18.66
	Day 4	18.19	17.93	17.44	16.78	18.02	18.15	17.38	16.73
	Day 7	16.99	16.56	16.47	16.58	17.26	17.48	16.83	15.73
Chroma value									
Low pHu	Day 1	20.53	23.39	22.88	22.68	22.42	22.61	21.69	22.82
	Day 4	17.63	19.69	19.05	19.79	18.03	19.4	17.59	20.19
	Day 7	17.24	18.74	17.51	19.01	17.48	17.41	16.99	18.6
Intermediate pHu	Day 1	22.23	23.04	21.62	22.02	22.98	21.85	23.02	20.49
	Day 4	19.8	19.92	18.96	18.02	19.83	19.34	19.76	17.96
	Day 7	18.5	18.36	16.76	17.74	18.48	17.5	18.26	15.96
High pHu	Day 1	20.48	20.72	19.3	19.97	20.16	20.91	20.18	19.24
	Day 4	18.9	18.88	17.97	15.86	18.37	18.65	17.77	16.44
	Day 7	17.08	16.33	16.39	15.55	16.97	17.5	16.5	14.73

AES, additional electrical stimulation; NAES, no additional electrical stimulation.

Low pHu (n = 3), Int (intermediate) pHu (n = 3), and high pHu (n = 6).

L^*^ = Treatment effect [AES vs NAES] (p>0.05); pH effect (p>0.05); **temperature effect (p<0.001)**; **day effect (p<0.001)**; interaction effect (stimulation×temperature) (p>0.05); (pH×temperature) (p>0.05); (pH×day) (p>0.05, SED = 1.79).

a^*^ = treatment effect [AES vs NAES] (p>0.05); **pH effect (p = 0.004)**; **temperature effect (p = 0.006)**; **day effect (p<0.001)**; interaction effect (stimulation×temperature) (p>0.05); **(pH×temperature) (p = 0.005)**; **(pH×day) (p = 0.004**, SED = 1.16).

b^*^ = treatment effect [AES vs NAES] (p>0.05); **pH effect (p = 0.022)**; **temperature effect (p = 0.003)**; **day effect (p<0.001)**; **interaction effect** (stimulation×temperature) (p>0.05); **(pH×temperature) (p = 0.017);** (pH×day) (p>0.05, SED = 0.63).

Hue angle = treatment effect [AES vs ES] (p>0.05); pH effect (p>0.05); **temperature effect (p = 0.005); day effect (p<0.001);** interaction effect (stimulation×temperature) (p>0.05); (pH×temperature) (p>0.05); **(pH×day) (p = 0.001**, SED = 1.28).

Chroma value = treatment effect [AES vs NAES] (p>0.05); **pH effect (p = 0.023);** temperature effect (p>0.05); **day effect (p<0.001); interaction effect (stimulation×pH) (p = 0.005)**; (stimulation×temperature) (p>0.05); **(pH×temperature) (p = 0.006); (pH×day) (p = 0.014**, SED = 1.25).
